# Supplementary material for: Formulation of Amphotericin B in PEGylated Liposomes for Improved Treatment of Cutaneous Leishmaniasis by Parenteral and Oral Routes
Source: Pharmaceutics. 2022 May 5;14(5):989. doi: 10.3390/pharmaceutics14050989 (PMC9147047; doi:10.3390/pharmaceutics14050989)
Supplement: Supplementary file 1 [file pharmaceutics-14-00989-s001.zip › pharmaceutics-1681125-supplementary.pdf]

Supplementary Materials

Formulation of amphotericin B in PEGylated liposomes for improved treatment of cutaneous leishmaniasis by parenteral and oral routes

By Guilherme S. Ramos, Virgínia M. R. Vallejos, Gabriel S. M. Borges, Raquel M. Almeida, Izabela M. Alves, Marta M. G. Aguiar, Christian Fernandes, Pedro P. G. Guimarães, Ricardo T. Fujiwara, Philippe M. Loiseau, Lucas A. M. Ferreira, and Frédéric Frézard

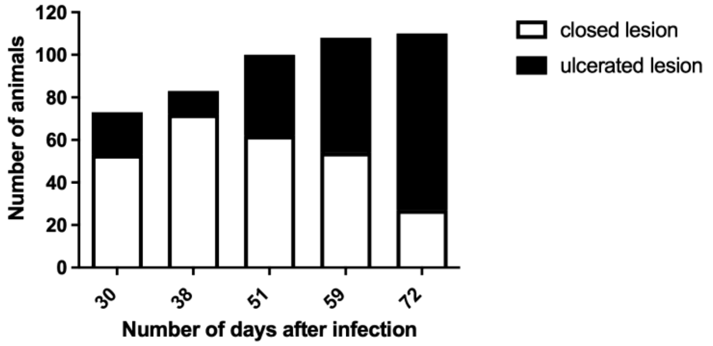

Figure S1. Time-course of appearance of closed and ulcerated lesions in BALB/c mice after infection with *Leishmania amazonensis* at the tail base. Data refers to animals used in the third *in vivo* experiment.

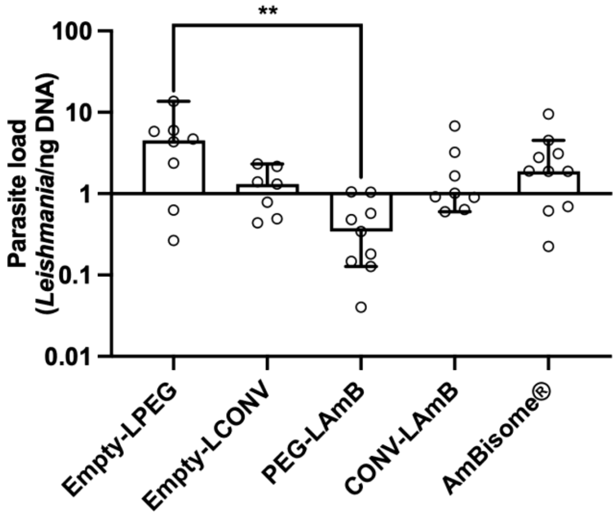

Figure S2. Parasite load in the spleen of mice with cutaneous leishmaniasis after treatment with conventional and PEGylated liposomal AmB by the IV route. BALB/c mice were infected intradermally with *Leishmania amazonensis*. Treatments were initiated 75 days after infection with 7 doses of the following formulations. CONV-LAmB group received the conventional liposomal AmB formulation at 5 mg/kg, every 4 days by IV route; PEG-LAmB group received the PEGylated liposomal AmB formulation at 5 mg/kg, every 4 days by IV route; AmBisome® group received AmBisome® at 5 mg/kg, every 4 days by IV route; Empty-LCONV group received by IV route empty conventional liposomal formulation at the same regimen (lipid dose, intervals) as that in CONV-LAmB group; Empty-LPEG group received by IV route empty PEGylated liposomal formulation at the same regimen (lipid dose, time intervals) as in PEG-LAmB group. Parasite load was determined in the spleen by qPCR. Data shown as medians + 95% confidence interval and analyzed by Kruskal-Wallis followed by Dunn's multiple comparison post-test. $^{**}p < 0.01$.

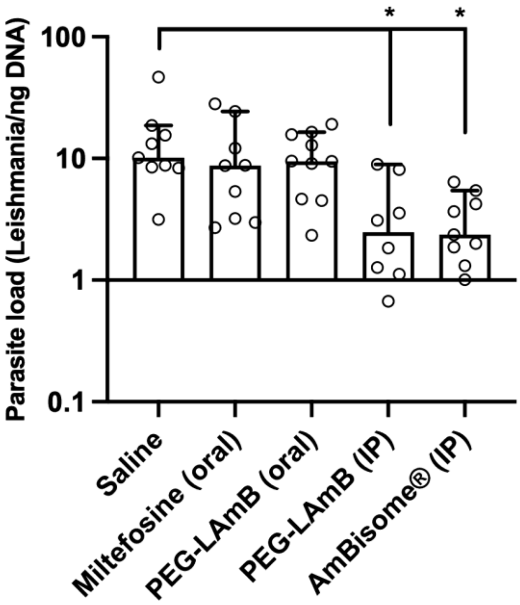

Figure S3. Parasite load in the spleen of mice with cutaneous leishmaniasis after treatment with PEGylated liposomal AmB by oral and IP routes. BALB/c mice were infected intradermally with *Leishmania amazonensis*. Treatments were initiated 75 days after infection with 10 doses of the following formulations. PEG-LAmB (oral) group received the PEGylated liposomal AmB formulation at 5 mg/kg, every 2 days by oral route; PEG-LAmB (IP) group received the PEGylated AmB liposomal AmB formulation at 5 mg/kg, every 2 days by IP route; AmBisome® (IP) group received AmBisome® at 5 mg/kg, every 2 days by IP route; Miltefosine group received miltefosine at 10 mg/kg, every 2 days by oral route; Saline group received isotonic saline by IP route. Parasite load was determined in the spleen by qPCR. Data shown as medians + 95% confidence interval and analyzed by Kruskal-Wallis followed by Dunn's multiple comparison post-test. *$p < 0.05$.

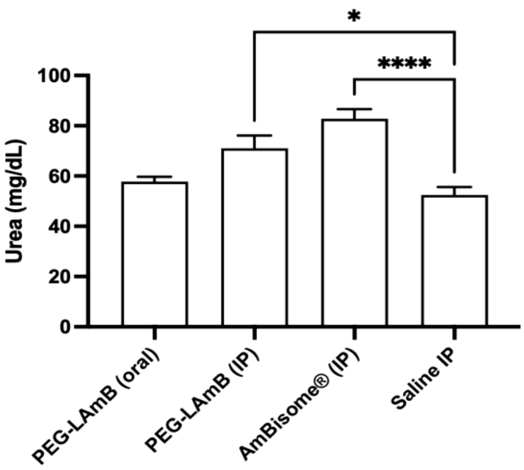

(**a**)

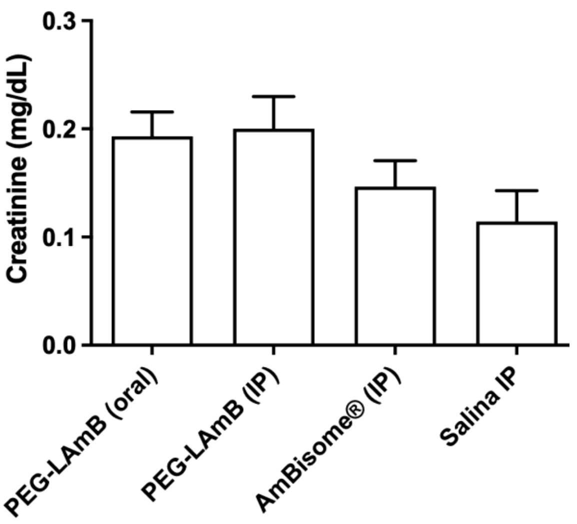

(**b**)

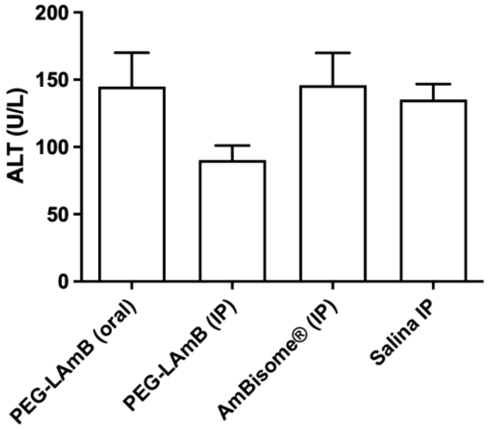

(**c**)

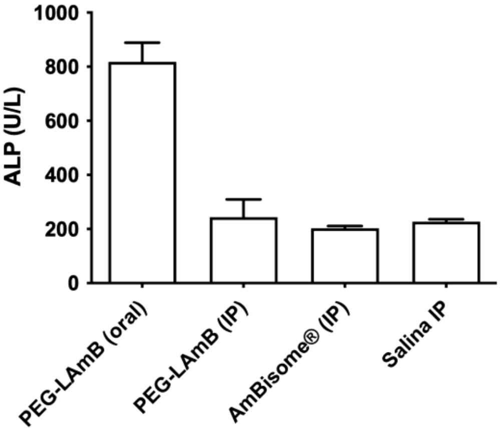

(**d**)

Figure S4. Plasma levels of (a) urea, (b) creatinine, (c) alanine aminotransferase (ALT) and (d) alkaline phosphatase (ALP) after treatment with different liposomal formulations of AmB. BALB/c mice were infected intradermally with *Leishmania amazonensis*. Treatments were initiated 75 days after infection with 10 doses of the following formulations. PEG-LAmB (oral) group received the PEGylated liposomal AmB formulation at 5 mg/kg, every 2 days by oral route; PEG-LAmB (IP) group received the PEGylated AmB liposomal AmB formulation at 5 mg/kg, on alternate days by IP route; AmBisome® (IP) group received AmBisome® at 5 mg/kg, every 2 days by IP route. Saline group received isotonic saline by IP route. Data is shown as means ± SEM. * $p < 0.05$, **** $p < 0.0001$ for statistical difference between the treated group and Saline group according to Kruskal-Wallis test with Dunn's multiple comparison post-test.

Table S1. Data of size characterization of the liposomal formulations [a]

CONV-LAmB		PEG-LAmB		AmBisome®		Empty-LCONV		Empty-LPEG	
Mean diameter (nm)	PI[b]	Mean diameter (nm)	PI[b]	Mean diameter (nm)	PI[b]	Mean diameter (nm)	PI[b]	Mean diameter (nm)	PI[b]
137.5	0.075	114.8	0.20	92.0	0.16	102.3	0.052	133.1	0.16
102.4	0.048	114.5	0.25	84.0	0.13	104.1	0.08	121.6	0.18
114.0	0.13	134.3	0.21	99.2	0.18	105.9	0.05	131.4	0.18
105.6	0.13	120.1	0.24	85.9	0.15	102.0	0.02	146.0	0.18
107.6	0.06	140.8	0.26	87.8	0.14	102.3	0.05	140.5	0.2
				86.9	0.14				
				82.0	0.14				
				95.5	0.18				

[a] used to generate data shown in Table 1; [b] PI: polydispersity index

Table S2. Data of zeta-potential (mV) of the liposomal formulations [a]

CONV-LAmB	PEG-LAmB	AmBisome®	Empty-LCONV	Empty-LPEG
-27.1	-3.2	-29.3	-27.8	-5.1
-27.1	-3.3	-29.5	-27.8	-4.6
-27	-3.7	-31.1	-28.8	-4.4

[a] used to generate data shown in Table 1

Table S3. Data of Area Under the Curve for determination of AmB by HPLC in the liposomal formulations[a]

Standard AmB	PEG-LAmB (before filtration)	PEG-LAmB (after filtration)	CONV-LAmB (before filtration)	CONV-LAmB (after filtration)
151647	143423	139858	135423	141485
155047	143111	139485	138111	143498
150000	142321	140468	136321	144925

[a]used to generate data shown in Table 1

Table S4. Data of parasite load in the lesion of *Leishmania amazonensis*-infected mice after different treatments[a]

Saline	Empty-Lipo (IP)	Miltefosine (oral)	CONV-LAmB (IP)	PEG-LAmB (IP)
3.951431e+007	2.728438e+007	982928.9	2.105986e+007	1.574929e+007
3.574781e+007	2.487186e+007	430305.7	7.758527e+007	6561562
7.262161e+007	9.43007e+007	35947.35	1.40213e+007	2099166
5.756177e+007	2.517152e+007	184114.2	3.263633e+007	3.410334e+007
6.194249e+007	1.625314e+007	2297877	1.15703e+008	3840997
1.492621e+008	6.025715e+007	61746.13	2.971452e+007	2649297
7.980959e+007		1875774	1.635823e+007	2.849393e+007
1.155948e+008		635285.6	4.002504e+007	1.896776e+007
3.951852e+007			4.595904e+007	

[a]used to generate graph shown in Figure 5(b)

Table S5. Data of lesion size variation in *Leishmania amazonensis*-infected mice during different treatments[a]

**Lesion size variation in Saline group**										
Initial size (mm)	12	9.5	10.75	11.5	10.5	9.5	10.75	11.5	7.5	11
Size variation										
Day 4	0.25	0.5	0.5	0.5	0	1	1	1.25	0.75	1.5
Day 8	1	2.5	2	1	1.25	3	0.25	3	2	2.5
Day 12	-1	1.5	-0.75	0	-0.5	1	0.75	1.5	1	1
Day 16	2.5	2.75	4.5	3.25	2	3.5	3.75	4.5	4.75	3.5
Day 20	5.5	4.75	7	6.5	3.75	5	5.75	6.75	7.25	5.5
Day 24	3	3.5	3.25	3	3	4	4.25	4.5	7	4
**Lesion size variation in Empty-lipo group**										
Initial size (mm)	12.25	10.25	13	10	7.5	9	9.75	6.25		
Size variation										
Day 4	1	1.75	-0.25	1.25	-0.25	1.75	1.75	2.5		
Day 8	2.75	2	2	2.75	1.75	1.5	2.75	3.75		
Day 12	-0.65	1	0	0.5	0.5	1	0.75	3.25		
Day 16	2.5	3.5	1.75	2	2.75	3.5	3.75	5.5		
Day 20	4.75	5.5	4.25	5	3.75	5.75	5	8.5		
Day 24	3	4.25	2.25	4.25	3	5.25	5.25	8.25		
**Lesion size variation in Miltefosine (oral)**										
Initial size (mm)	8.5	12.25	11	10.75	9.75	10	11.25	8.25	8	
Size variation										
Day 4	0.5	-0.25	-0.75	0.75	0.25	1	-0.25	0.25	-0.25	
Day 8	0.25	-0.5	-1.25	-0.75	-1.25	-1.5	-0.25	0	0.75	
Day 12	-2.5	-4.25	-4	-4	-3.25	-4.75	-4.75	-1.75	-1.75	
Day 16	-0.75	-3.5	-1,5	-3.75	-1.75	-2.5	-2	-0.5	0	
Day 20	-2.25	-2.5	-3.75	-3.25	-2.5	-2.5	-4.25	-1.25	-1.75	
Day 24	-8.5	-12.25	-11	-10.75	-9.75	-10	-11.25	-8.25	-8	
**Lesion size variation in CONV-LAmB (IP)**										
Initial size (mm)	12.75	11	10.25	12.5	11.25	10.5	9.25	8.5	7.75	5.5
Size variation										
Day 4	0.25	0	0.75	0.75	2	0.75	2.25	1.25	1.75	3.25
Day 8	0.25	0.5	1.75	0.75	-0.75	1.25	2.25	2	2.25	2.75
Day 12	1.5	0.75	0.25	0.75	-0.25	1.5	2.5	1.5	0	1
Day 16	2	3	2.25	1.5	3.25	4.75	5.5	3.25	5.25	4.25
Day 20	4.5	4.5	3.75	4	5.25	4.5	7	5	7.25	6.75
Day 24	2.75	4.75	2.75	3.5	4.25	4	4	4.25	6.5	4.25
**Lesion size variation in PEG-LAmB (IP)**										
Initial size (mm)	9.5	11.5	11.25	10.5	8	9.75	9.5	9	7.75	
Size variation										
Day 4	1	0.75	1	2	1	1	0.75	0.25	0	
Day 8	1.75	2.5	1	2.25	1.25	1.75	1	1.25	0.25	
Day 12	-0.75	0.25	-1.5	0.25	0.5	0	-1	-0.75	-0.75	
Day 16	2	1.25	0	2	1.75	1.5	1	0	1.25	
Day 20	35	2.5	1.5	3	3.25	2	0.75	2.75	2.5	
Day 24	2.5	1.25	0.25	2.25	1	0.75	1.25	1.25	2.75	

[a]used to generate graph shown in Figure 5(a)

Table S6. Data of parasite load in the lesion of *Leishmania amazonensis*-infected mice after different treatments[a]

PEG-LAmB (IV)	CONV-LAmB (IV)	AmBisome® (IV)	Empty-LPEG (IV)	Empty-LCONV (IV)
3119210	1518040	3936614	3320385	6537194
1314083	9284643	760072.6	3201206	1388397
454217.8	839240.9	1876434	1.1999205e+007	713175.1
597441.5	3485429	1511863	6159556	6931697
565137.6	2024532	704088.1	9797289	107704
663080.5	661457.8	9603377	2027968	6318406
2158520		4132696	1295599	1.3252417e+007
701958.6		1019607	3639428	
		5339034		
		3250016		

[a]used to generate graph shown in Figure 5(d)

Table S7. Data of lesion size variation in *Leishmania amazonensis*-infected mice during different treatments[a]

**Lesion size variation in PEG-LAmB (IV)**										
Initial size (mm)	12.5	10	12.5	9.5	12.25	5.25	7.25	8.5		
Size variation										
Day 7	1.75	1.25	-0.25	0.5	1	1.25	0	1.5		
Day 14	1	2.75	2	2.5	2.25	2.5	1.75	2.75		
Day 21	1	3.25	2.25	2.75	2.5	3	1.5	3.75		
Day 28	0	4.25	1	2.75	2.5	3	2	3.75		
**Lesion size variation in CONV-LAmB (IV)**										
Initial size (nm)	12	12.25	13.25	11.75	13	4.75	6.25	9		
Size variation										
Day 7	1	1.25	1.5	1.25	2.25	0	1	1.75		
Day 14	2.5	2.75	0.75	2.25	3	0.75	2.25	3.75		
Day 21	3.25	3.75	0.75	3	3	0.75	2.75	4.25		
Day 28	3.25	2.25	1.5	4	4	1.25	3.5	5		
**Lesion size variation in AmBisome® (IV)**										
Initial size (mm)	11.25	9.25	12.5	14	10	9.25	7.5	8	9	5.5
Size variation										
Day 7	0.75	1.75	2.75	1.75	0.5	1	0.75	2.5	-0.5	0
Day 14	2	1.5	5.25	3	2	2.75	1	3.75	4.75	2.75
Day 21	2.25	2.25	5.25	3	2.5	4	1	5.25	6	4.75
Day 28	2.5	3.75	5.75	3.5	2.5	3.5	1.25	5.5	5.5	5
**Lesion size variation in Empty-LPEG (IV)**										
Initial size (mm)	8.5	13.75	11.75	8.75	13.25	6.75	10.5	8		
Size variation										
Day 7	1.25	3.25	1.25	2.25	1	1	2	1		
Day 14	2.25	3	1.75	3	1.25	2	3.75	3.25		
Day 21	2.5	2.5	3.25	4.25	2.5	2.25	6.5	5.5		
Day 28	4	3.25	4.25	4.75	2.5	3.25	7.25	6.75		
**Lesion size variation in Empty-LCONV (IV)**										
Initial size (mm)	8.25	13.5	13.25	9.5	9.25	10	8			
Size variation										
Day 7	0.75	2.5	1.8	1	2	2	3			
Day 14	2	3.75	2.55	3	4	4.25	3.5			
Day 21	3	4.75	4.3	5	5	4.5	4.75			
Day 28	3	4	4.55	6	5.75	5.75	5.75			

[a]used to generate graph shown in Figure 5(c)

Table S8. Data of parasite load in the lesion of *Leishmania amazonensis*-infected mice after different treatments[a]

PEG-LAmB (oral)	PEG-LAmB (IP)	AmBisome® (IP)	Miltefosine (oral)	Saline
4448969	3958086	2.3156338e+007	2052225	7.1102781e+007
3.5210932e+007	9340393	8659047	1.0000894e+007	5.1600708e+007
2.1130283e+007	272440.6	1.9168005e+007	2.9374174e+007	1.5081568e+007
2.8910086e+007	1323977	3.0511656e+007	2.3334239e+007	1.5536152e+007
1.325993e+007	6088030	1.4545843e+007	232756.7	2.7914494e+007
9649317	6715694	2979606	107408.1	2.1199382e+007
1.4779308e+007	52070,2	4.4545516e+007	3565089	6.1024524e+007
5442365	5307713	660162.6	1117244	2.6586141e+007
4092031	1580392	1.5185133e+007	1.5297303e+007	1.4419335e+007
1.0982103e+007		1.9444747e+007		

[a]used to generate graph shown in Figure 6(b)

Table S9. Data of lesion size variation in *Leishmania amazonensis*-infected mice during different treatments[a]

**Lesion size variation in PEG-LAmB (oral)**										
Initial size (mm)	13	14.75	11.25	12.5	13.75	12.5	8.25	4.75	11.75	12.5
Size variation										
Day 7	1	0	1.25	1.5	0.75	0.5	3.75	1	-0.25	1
Day 14	1	0.75	1.5	2	0.75	-0.25	4.75	0.75	0	1.75
Day 21	1.5	1.75	1.75	2	0.75	0	5	1.75	0	2
Day 27	1.75	1.75	2	2	1.5	1.25	5.75	2	1.25	2.55
**Lesion size variation in PEG-LAmB (IP)**										
Initial size (mm)	12.75	9.2	11.75	8.75	12	13.75	14.25	12.5	13	6.5
Size variation										
Day 7	-0.25	1.05	0	0.25	0	0.25	1.25	1	0	0.25
Day 14	0.25	0.8	0.25	-0.5	-0.25	0	0.75	0.75	-0.5	0.25
Day 21	0.25	0.8	0	-0.75	0	-0.25	-1.25	-5.75	-0.5	6
Day 27	1.5	2.05	0.25	-1.25	1.25	0.5	-1.25	0	0.25	0.5
**Lesion size variation in Miltefosine (oral)**										
Initial size (mm)	8	16.25	9.75	11	10.75	14	7.75	12	11.25	13
Size variation										
Day 7	1.25	0	0.75	0.25	0.25	1	0.25	1	0.75	1
Day 14	1.25	-0.5	0.5	0.5	-0.25	1	0.25	0.5	0.25	1.5
Day 21	0	-1.25	0	1.75	-0.75	0.25	0	0.5	0	1.5
Day 27	-0.25	-2	-0.25	1.5	-1.25	0	-0.25	0.5	-0.5	1.5
**Lesion size variation in AmBisome® (IP)**										
Initial size (mm)	12.75	11.5	12.25	13.25	12.25	6.25	13	9.5	10.25	10
Size variation										
Day 7	0.5	0.5	-1.25	0.75	0.25	0.25	-0.25	-1	0.25	0.25
Day 14	0.5	0.75	-1.5	1	0.75	0.25	1	-1.5	1.25	0.75
Day 21	0.75	1.25	-1	0.75	1	0.25	1	-1.5	1.25	1
Day 27	2.75	1.75	-0.5	0.5	1.25	0.5	2.25	-0.75	2.25	2.25
**Lesion size variation in Saline (IP)**										
Initial size (mm)	12.75	13	12	9.75	7.75	8.75	12.25	9.75	12	10
Size variation										
Day 7	-1	0.75	0.5	0.5	0.25	1.25	0.25	1	0.75	0
Day 14	0.5	2.25	2	1.25	2.5	3	2	3	2	2.25
Day 21	2.5	3.5	3.5	2	4.25	4.25	3.75	5.5	4.25	5.25
Day 27	4	5.25	5	4	4.25	5.75	4.75	6	4.5	5

[a]used to generate graph shown in Figure 6(a)
